# Inadvertent Central Venous Catheter Placement in the Vertebral Vein: A Case Report

**DOI:** 10.7759/cureus.105683

**Published:** 2026-03-23

**Authors:** Amr Khorkhash, Ahmad Zahran

**Affiliations:** 1 Emergency Medicine, East and North Hertfordshire NHS Trust, Stevenage, GBR; 2 Diagnostic and Interventional Radiology, Ain Shams University Hospitals, Cairo, EGY

**Keywords:** catheter-associated thrombosis, central venous access, high-resolution ct scan, minimally invasive interventional radiology, ultrasound-guided

## Abstract

Central venous catheter (CVC) placement is a common procedure in the hospital setting, with large numbers of patients undergoing central venous catheterisation every year worldwide. Central venous catheterisation is recommended to be done under real-time ultrasound guidance to decrease procedure-related complications. Nevertheless, complications are not infrequently encountered. One of the risky complications of central venous catheterisation is CVC misplacement. Here, we present an unusual case of inadvertent left vertebral vein central venous catheterisation and describe a simple method of management.

## Introduction

Central venous catheterisation is widely used in modern-day clinical practice, with large numbers of patients receiving central venous catheterisation yearly [1]. Common complications associated with central venous catheter (CVC) placement include pneumothorax, haematomas, inadvertent arterial puncture, pseudoaneurysms, arteriovenous fistulas (AVFs), arterial dissection, haemothorax, catheter malposition, lymphatic duct injury, deep venous thrombosis, and bloodstream infections [2]. The use of real-time ultrasound guidance significantly decreases the rate of mechanical complications during CVC placement [2]. It is recommended to place all CVCs under real-time ultrasound guidance to decrease the risks associated with CVC placement [3]. Despite the widespread use of ultrasound in CVC placement, technical complications are still encountered in clinical practice. We report an unusual case of a misplaced CVC in the left vertebral vein despite the use of ultrasound guidance.

## Case presentation

A 14-year-old male patient was admitted to our paediatric surgery department at Ain Shams University Hospitals for emergency explorative laparotomy for suspected intestinal obstruction. He had mild leucocytosis, and within normal haemoglobin, platelet count, prothrombin time, partial thromboplastin time, and international normalised ratio (exact laboratory values were not retrievable due to a hospital information system failure; however, clinical documentation indicated normal coagulation parameters at the time of catheter placement).

He underwent intraoperative ultrasound-guided central venous catheterisation to manage haemodynamics and to aim for better resuscitation, including the administration of emergency drugs. The central venous catheterisation procedure was described as uneventful, with venous blood aspiration from the first trial and subsequent smooth guidewire and CVC introduction.

**Figure 1:**
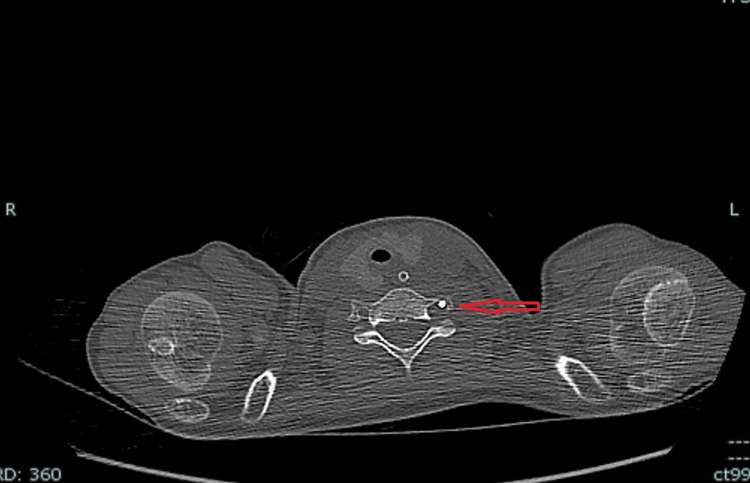
Computed tomography showing the central venous catheter (red arrow) passing through the left transverse foramen of C7.

The post-surgical course of the patient was complicated by pneumonia, for which he underwent computed tomography (CT) examination of the chest on postoperative day four. CT examination accidentally demonstrated the CVC passing through the transverse foramen of the left C7 cervical vertebra with its tip terminating in the left brachiocephalic vein (Figure 1).

**Figure 2:**
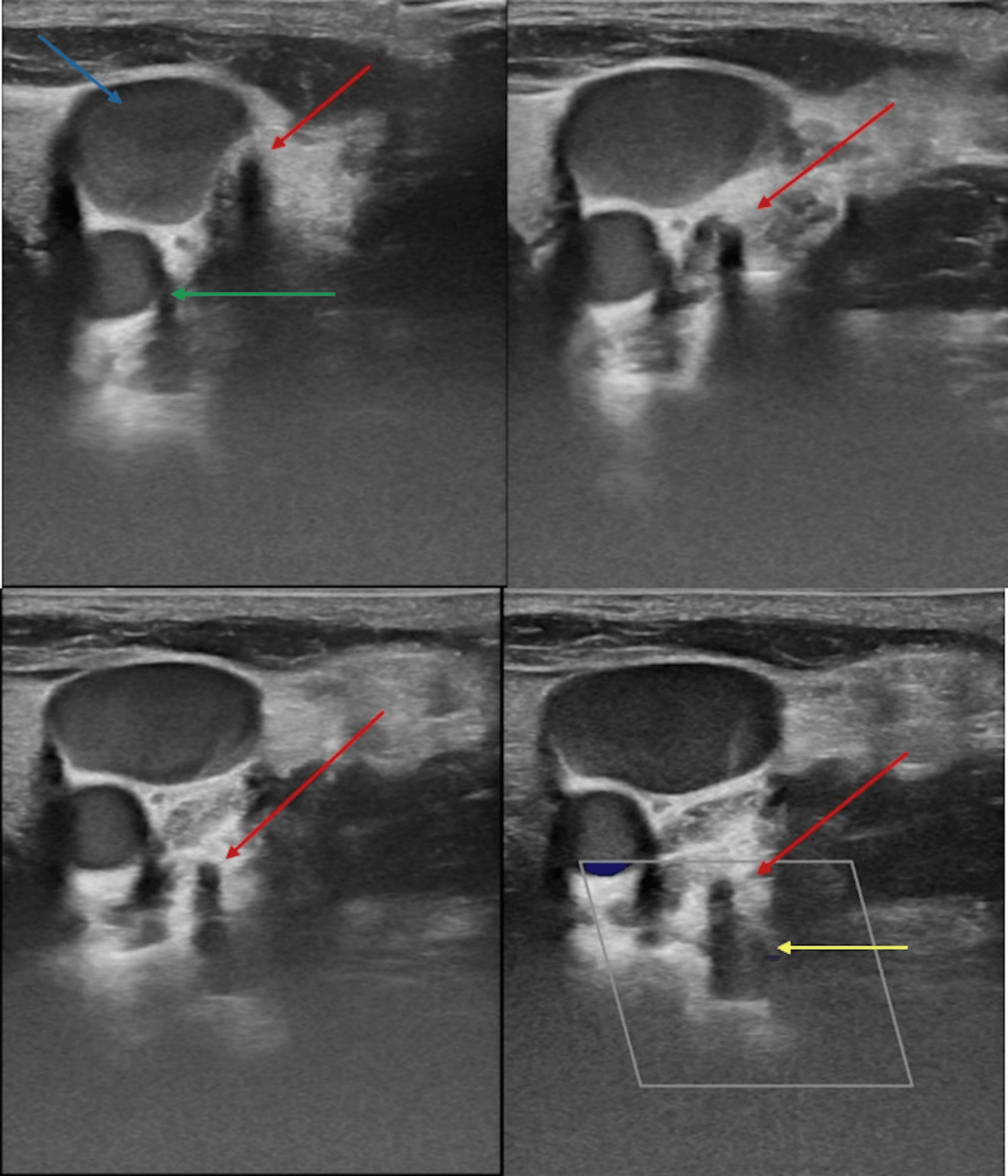
Serial ultrasound images depicting the central venous catheter (red arrow) showing posterior acoustic shadowing, coursing just posterior to the internal jugular vein (blue arrow) down to the vertebral vein and superficial to the vertebral artery (yellow arrow). The common carotid artery is also seen (green arrow).

**Figure 3:**
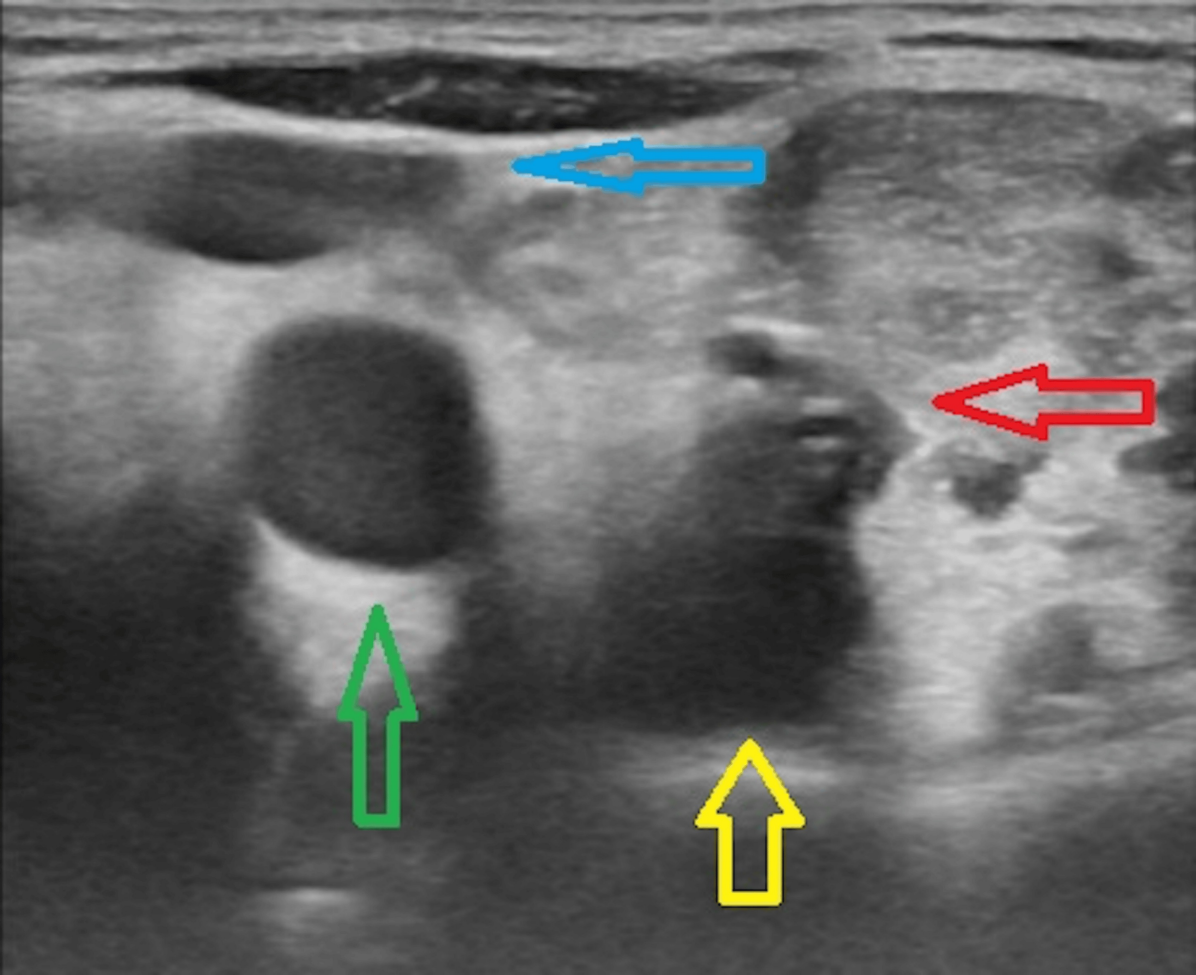
The central venous cathether is clearly seen as a circular echogenic structure inside the vertebral vein (red arrow) surrounded by an echogenic thrombus. It is seen lying superficial to the vertebral artery (yellow arrow) and medial to the internal jugular vein (blue arrow) and common carotid artery (green arrow).

We were consulted by the paediatric surgery department to remove the vertebral vein CVC and assess for potential complications. We planned for CVC removal and considered removing it over a safety guidewire in the Angio suite and performing vertebral artery angiography to exclude vertebral AVF. However, we were able to track the catheter course by ultrasound in the vertebral vein superficial to the vertebral artery (Figure 2). Ultrasound examination showed segmental thrombosis of the vertebral vein (Figure 3), and Doppler examination showed normal venous waveforms across the patent segments of the vertebral vein with no evidence of AVF. We also noted that the CVC entered the vertebral vein at C7 transverse foramen, the vertebral artery enters the transverse foramina at the level of C6, making it less likely to be injured at the C7 transverse foramen level.

We proceeded with bedside removal of the CVC in the intensive care unit without angiography and achieved haemostasis by ultrasound-guided compression for five minutes. Post-compression Doppler examination showed normal waveforms across the vertebral artery and normal venous low-velocity waveforms inside patent segments of the vertebral vein, with no haematomas detected.

We initiated a therapeutic dose of anticoagulation with low-molecular-weight heparin for the segmental thrombosis of the left vertebral vein. We then placed a right internal jugular vein CVC under ultrasound guidance. A follow-up vertebral arterial and venous Doppler examination was performed after 24 hours and still showed no evidence of AVF. The patient showed no neurological deficits during his stay at the hospital, and he was discharged two weeks later in good condition.

## Discussion

Inadvertent vertebral venous access is an exceedingly rare complication of central venous catheterisation with only a few cases reported in the literature [4,5]. Although very rare, vertebral artery injury or dissection can occur if mistakenly punctured during CVC placement [6]. Vertebral artery dissection can lead to a catastrophic posterior circulation stroke [7].

Magro et al. studied the vertebral venous plexus anatomy both in cadaveric specimens and in healthy adults. They found that in the majority of cases, the vertebral venous plexus starts from the skull base down to the C6 transverse foramen level, where it emerges as a single draining vein (the vertebral vein) that lies ventral and lateral to the vertebral artery and descends down to end at the brachiocephalic vein [8]. Because it has an almost overlapping course with the internal jugular vein and terminates in the brachiocephalic vein, misplaced CVC catheters in the vertebral vein can look very similar to an internal jugular vein catheter on a plain chest X-ray.

Ito et al. described a similar case, where a CVC was mistakenly placed in the right vertebral vein at the level of C6, necessitating removal in the catheterisation laboratory and undergoing diagnostic cerebral angiography to exclude a vertebral artery injury [4].

We demonstrated the efficacy of ultrasound in our case, assisting in the diagnosis, planning, and management of this complication. Ultrasound clearly depicted the course of the CVC in the vertebral vein below the C7 transverse foramen level, with the vein seen superficial to the vertebral artery. It showed segmental thrombosis of the vertebral vein at the site of entry, making bleeding after removal less likely. The vertebral vein puncture site was at the level of the C7 transverse foramen, while the vertebral artery lies outside the foramen, making it less likely to be injured. Furthermore, we used ultrasound-guided compression to detect any bleeding early on before any further sequelae occurred.

Luckily, no vertebral artery injury or major complications occurred in our patient; however, similar complications emphasise the importance of using good ultrasound technique and ensuring proper visualisation of the needle along its path to the targeted vein, as well as demonstration of the guidewire inside the target vein before tract dilatation and CVC placement. Several factors may have contributed to the misplacement of the CVC into the vertebral vein despite the use of ultrasound guidance, including operator experience, as it was placed by a resident; second, failure to examine the guidewire course before resuming with dilatation and CVC placement, as this step is very crucial in preventing CVC misplacement.

## Conclusions

Vertebral vein malposition of a CVC is an uncommon but important complication that may be radiographically occult. Ultrasound plays a critical role not only in preventing malposition but also in diagnosing and safely managing it when it occurs. In carefully selected, stable patients, detailed sonographic assessment of catheter course and vertebral artery integrity may allow safe bedside removal without angiography. Recognition of cervical vascular anatomy, particularly the relationship between the vertebral vein and vertebral artery at the C6-C7 level, is essential for both prevention and management. This case highlights the importance of meticulous ultrasound-guided technique and post-placement vigilance to reduce avoidable morbidity.
